# Achromatic 3D Multi‐Color Orbital Angular Momentum Holography

**DOI:** 10.1002/advs.202503488

**Published:** 2025-04-24

**Authors:** Hang Su, Baoli Li, Yike Bai, Yiqi Ye, Yibo Dong, Xinyuan Fang

**Affiliations:** ^1^ School of Artificial Intelligence Science and Technology University of Shanghai for Science and Technology Shanghai 200093 China; ^2^ Institute of Photonic Chips University of Shanghai for Science and Technology Shanghai 200093 China

**Keywords:** 3D display, orbital angular momentum, two‐photon lithography, wavelength‐multiplexed holography

## Abstract

Orbital angular momentum (OAM) holography represents a transformative technique for enhancing the information channel capacity of holographic systems through the convolution of theoretical infinite OAM states with discrete sampling target images. However, the inherent axial chromatic aberration (ACA) in Fourier lens functions leads to the misalignment of Fourier planes for OAM‐dependent holograms across different wavelengths, thereby limiting 3D multicolor OAM holography. To address this challenge, a novel approach achieving achromatic 3D multi‐color OAM holography using a spatial multiplexing scheme is presented. Specifically, an optically digitalized achromatic OAM‐dependent hologram is developed by superposing wavelength‐specific lens functions onto monochromatic Fourier OAM‐dependent holograms, followed by high‐resolution spatial discretization and interleaving via two‐photon lithography (TPL). Experimental results demonstrate that the method enables a double‐plane (along the propagation direction of light), 3‐color (633, 532, and 460 nm), and 3‐channel OAM‐multiplexing holographic display (OAM order *l*_en_ = 1,4, and 7) with the ACA correction, utilizing a nano‐printed hologram featuring a subwavelength (600 nm) pixel size. This breakthrough paves the way for high‐capacity, multi‐spectral 3D holographic data storage and retrieval, with significant implications for augmented reality (AR)/virtual reality (VR) display, optical holographic encryption, and optical artificial intelligence.

## Graphical abstract

This work presents an achromatic 3D multicolor orbital angular momentum (OAM) holography based on spatial multiplexing. By integrating wavelength‐specific lens functions onto monochromatic Fourier OAM‐dependent holograms and high‐resolution spatial discretization and interleaving via two‐photon lithography (TPL), the approach addresses axial chromatic aberration‐induced misalignment in Fourier planes across wavelengths and enables precise reconstruction of 3D multicolor OAM holograms.

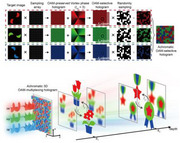

## 1 Introduction

Holography involves using a hologram to record and reconstruct the 3D wavefront of objects,^[^ ^1^ ^]^ which has been widely applied in 3D displays,^[^ ^2^ ^]^ optical encryption,^[^ ^3^ ^]^ data storage,^[^ ^4^ ^]^ and artificial intelligence.^[^ ^5^ ^]^ With the dramatic increase of the amount of information in the era of big data, various physical dimensions of light, such as wavelength,^[^ ^6^, ^7^ ^]^ polarization,^[^ ^8^, ^9^, ^10^, ^11^ ^]^ angle of incidence,^[^ ^12^ ^]^ space^[^ ^13^ ^]^ and time,^[^ ^14^, ^15^ ^]^ have been adopted as information carriers in holography to improve the information capacity of a single hologram. The resultant concept of optical multiplexing holography^[^ ^16^ ^]^ has witnessed great success in numerous applications including high‐security optical encryption,^[^ ^17^, ^18^ ^]^ optically addressable dynamic display,^[^ ^19^ ^]^ and parallel neuromorphic optical computing.^[^ ^20^ ^]^ In this regard, the improvement of the information channel is a great demand.

Orbital angular momentum (OAM), represented by a helical wavefront, exp(*jlφ*) (where *l* and *φ* denote the helical mode index and the azimuthal angle of a helical wavefront, respectively), has attracted significant attention in information optics due to its theoretically unbounded helical mode index,^[^ ^21^, ^22^, ^23^, ^24^ ^]^ greatly facilitating pioneering concepts such as high‐capacity optical communication,^[^ ^25^ ^]^ 6D data storage,^[^ ^26^ ^]^ spatiotemporal light fields,^[^ ^27^ ^]^ and high‐dimensional quantum entanglement.^[^ ^28^ ^]^ Recently, the concept of OAM multiplexing has been extended to the field of optical holography.^[^ ^29^ ^]^ Specifically, through appropriate spatial frequency sampling of a digital hologram in momentum space, the Fourier transformation of a discretized target object is convoluted with an OAM helical wavefront to create an OAM‐dependent hologram. To further increase the information channel number in practical applications, researchers have begun to focus on the synergistic multiplexing of OAM and the other physical dimensions. For example, the light beams with distinctive spin eigenstates and OAM states can be individually encoded in a high‐security nested holographic encryption scheme.^[^ ^30^ ^]^ Additionally, a pseudo‐incoherent approach has been provided to improve the spatial resolution of the holographic images in OAM holography through temporal multiplexing.^[^ ^31^ ^]^ Most recently, wavelength, which plays a crucial role in human visual perception, has also been explored to obtain multi‐color OAM holography.^[^ ^32^, ^33^, ^34^ ^]^ However, due to the axial chromatic aberration (ACA) caused by the dispersion of lens functions for Fourier transformation,^[^ ^35^ ^]^ the reconstructed images at distinct wavelengths cannot be achieved in the same Fourier plane, hindering the development of 3D colorful holography.

As a direct laser writing nano‐fabrication technology, two‐photon lithography (TPL) has revolutionized the field of holographic device manufacturing, offering unprecedented precision in the fabrication of 3D structures.^[^ ^36^ ^]^ Compared to traditional electron beam lithography^[^ ^37^ ^]^ and focused ion beam lithography,^[^ ^38^ ^]^ TPL stands out for its ability to create complex volume holographic devices and polarization‐independent holographic devices, thanks to its remarkable ability of customizing arbitrary 3D shapes with a sub‐100 nm spatial resolution on universal substrates.^[^ ^39^, ^40^ ^]^ These advancements pave the way for the development of innovative 3D nano‐printed metasurfaces, which leverage the height of meta‐atoms as an additional degree of freedom to address the critical challenge of broadband imaging.^[^ ^41^ ^]^ Furthermore, the integration of random interleaving sub‐holograms of three distinct wavelength channels (red, green, blue) has facilitated the fabrication of holographic broadband diffractive meta‐lenses that support achromatic imaging,^[^ ^42^ ^]^ as well as an on‐chip achromatic holographic OAM mode detector at three wavelengths.^[^ ^43^ ^]^

Here, we demonstrate achromatic 3D multi‐color OAM holography by implementing Fourier transform with wavelength‐specific lens functions. Our method enables precise spatial coincidence control across multiple wavelengths in the spatial frequency domain, thereby facilitating wavelength‐ and OAM‐multiplexing holography in 3D space with ACA correction. Our approach begins with the design of an achromatic OAM‐selective hologram, achieved through spatial discretization and interleaving of monochromatic OAM‐selective holograms at three distinct wavelengths (460, 532, and 633 nm). This achromatic OAM‐selective hologram system allows for multi‐wavelength imaging at the same spatial position while maintaining OAM selectivity. By further leveraging TPL, we fabricated the optically digitalized achromatic OAM‐multiplexing hologram with a spatial resolution of 600 nm, successfully multiplexing achromatic OAM‐selective holograms encoded in three OAM channels (*l_en_* = 1, 4, 7) across two distinct imaging planes. Experimental results demonstrate the feasibility of this approach, with reconstructive colored images achieving an average structural similarity index (SSIM) of ≈0.74. Our work paves the way for compact, high‐capacity 3D color holographic systems, with potential applications in augmented reality (AR)/virtual reality (VR) display,^[^ ^44^, ^45^ ^]^ optical holographic encryption,^[^ ^3^, ^46^ ^]^ and optical artificial intelligence.^[^ ^5^, ^47^, ^48^ ^]^

## 2 Results and Discussion

### 2.1 Axial Chromatic Aberration of Lens Functions

**Figure 1:**
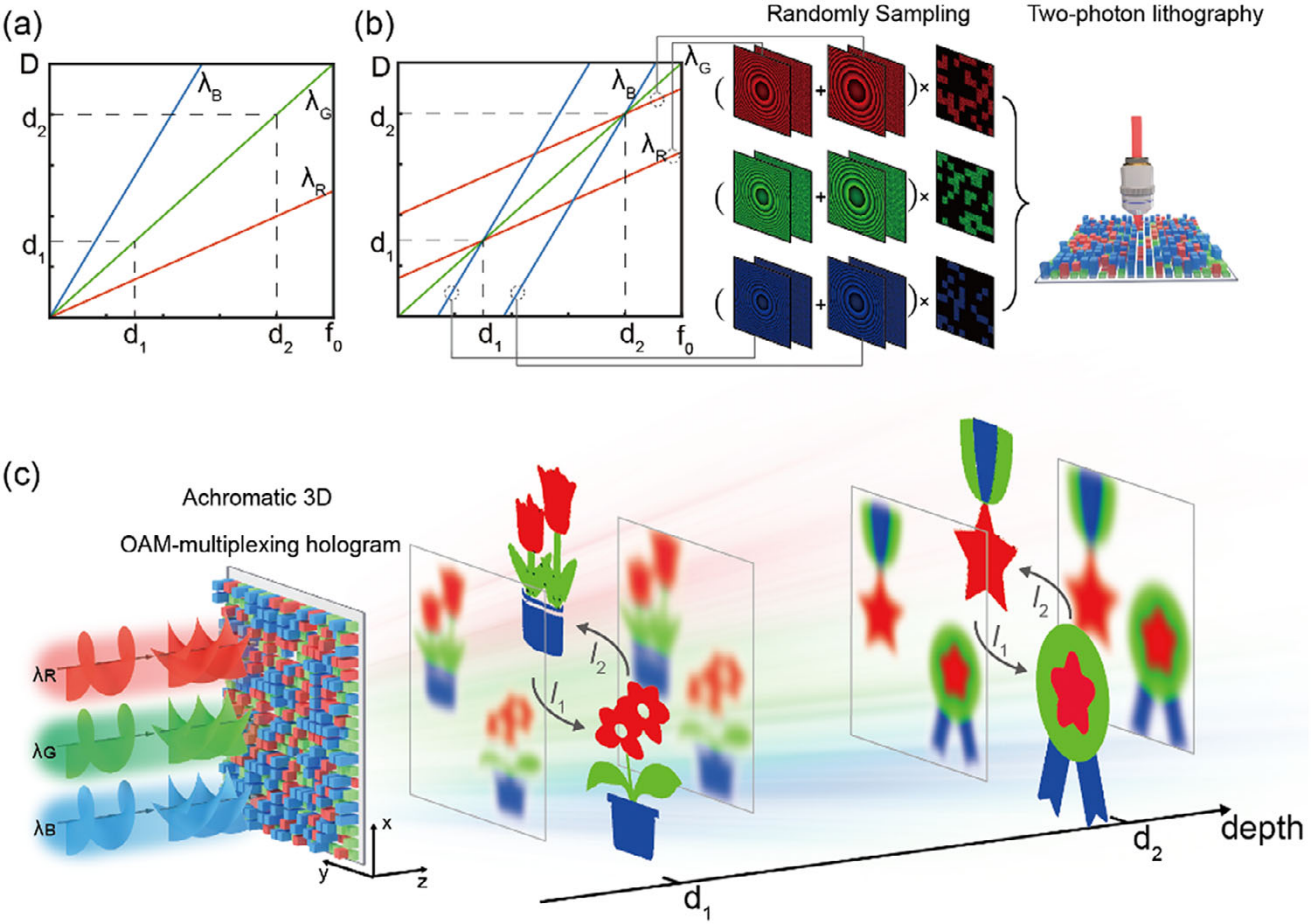
Conceptual illustration of achromatic 3D OAM‐multiplexing holography with ACA correction. a) Wavelength‐dependent shift of holographic imaging planes due to the ACA of Fourier lens function. b) Correction of the ACA based on spatial multiplexing scheme. c) Schematic illustration of the multi‐wavelength, OAM‐switchable, double‐plane holographic displays.

The depth of the holographic imaging plane (*D*) in Fourier holography is fundamentally determined by the lens function for Fourier transformation. Specifically, the ACA of lens functions causes a wavelength‐dependent spatial shift of the reconstructed images along the propagation direction of light, as illustrated in **Figure** **1**a, the slope of the line is *λ*_G_ */λ*_0_, representing the ACA of different wavelengths. To quantify this effect, we analyze the ACA of Fourier lens functions, which is characterized by the wavefront distortions introduced by the lens. The phase profile of a lens function, *t*_1_ *(x,y)*, is governed by Equation. (1) below,

(1) $$t_{1}\left(x,y\right)=\exp\left[-j\frac{k}{2f}\left(x^{2}+y^{2}\right)\right]=\exp\left[-j\frac{\pi }{f\lambda }\left(x^{2}+y^{2}\right)\right]$$

where *k* is the wavevector, *λ* is the wavelength of incident light, and *f* is the pre‐set focal length of the lens. For a lens that provides a focal length of *f*_0_ operating at wavelength *λ*_0_, the focal length for red light *λ*_R_ is given by *f*_R_ *= λ*_0_ */λ*_R_ *f_0_, which demonstrates that the ACA becomes more pronounced as the pre‐set focal length increases. This wavelength‐dependent shift introduces a fundamental limitation in the Fourier holographic imaging systems, particularly when reconstructing images across OAM beams with multiple wavelengths.

To address this challenge, we employ wavelength‐specific lens functions, each optimized for a particular wavelength, to achieve precise spatial coincidence of the focal planes across multiple wavelengths. As shown in Figure 1b, the longitudinal intercept is *d*_i_ *(λ_0−_ *λ_G_)/λ*_0_, and the focal lengths of six distinct lens functions are constrained to be *d*_1_ and *d*_2_ for the light beams with wavelengths of *λ_G_*, *λ_R,_* and *λ_B_*. Subsequently, the complex superposition of the phase profiles at the same wavelengths is implemented, followed by random spatial discretization and interleaving via high‐resolution TPL. This approach enables the correction of the ACA, thereby achieving achromatic 3D OAM‐multiplexing holography for multi‐wavelength, OAM‐switchable, double‐plane holographic displays (Figure 1c). As can be seen, the color images can be reconstructed with high fidelity at specific propagation distances (*D* = *d*_1_, *d*_2_) from the ultra‐compact optically digitalized hologram.

### 2.2 Design Principle of the Achromatic OAM‐Selective Hologram

**Figure 2:**
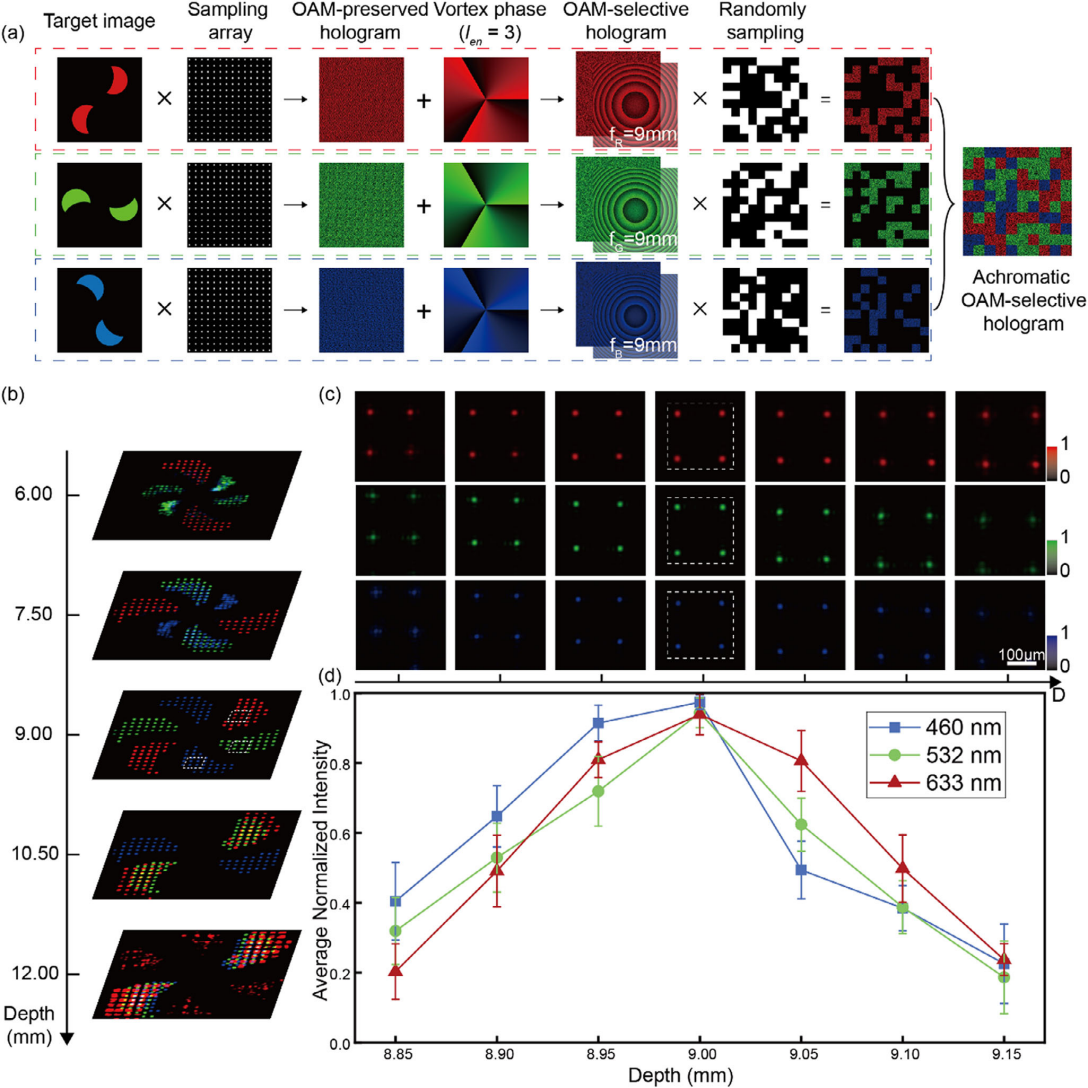
Design principle of the achromatic OAM‐selective hologram. a) Design framework of achromatic OAM‐selective hologram. b) Simulated reconstructed images from the achromatic OAM‐selective hologram at various depths. c) Normalized intensity distributions of the four selected pixels of the images encoded in each wavelength channel across different depths. d) Numerical analysis of the average normalized intensities of the four selected pixels in each wavelength channel across different depths.

To achieve achromatic OAM‐selectivity in multi‐color holography, we present a novel design framework of hologram as illustrated in **Figure** **2**a. The process begins with the iterative Fourier transform of discretely sampled target images, yielding monochromatic OAM‐preserved holograms at different wavelengths, the sampling distance of the array is shown in Supplementary Note 1 and Supplementary Figure S1 (Supporting Information). Next, to achieve OAM selectivity in holography, the helical phase plates with *l_en_* = 3 were encoded onto the monochromatic OAM‐preserved holograms, resulting in the monochromatic OAM‐selective holograms at specific wavelength channels. Notably, to achieve precise spatial coincidence of the holographic imaging planes across these wavelengths, we implement a random spatial discretization and interleaving scheme on the monochromatic OAM‐selective holograms encoded with wavelength‐dependent lens functions (Supplementary Note 2 and Supplementary Figure S2, Supporting Information), this process can be described by Equations. (2) and (3):

(2) $$H^{\prime }\left(x,y\right)=H\left(x,y\right)\cdot \exp\left(-j\frac{k}{2f}\left(x^{2}+y^{2}\right)\right)$$

(3) $$H_{m}\left(x,y\right)=H_{R}^{\prime }\left(x,y\right)\cdot S_{1}+H_{G}^{\prime }\left(x,y\right)\cdot S_{2}+H_{B}^{\prime }\left(x,y\right)\cdot S_{3}$$

where, *H(x,y)* represents the OAM‐selective hologram, and *S*_1_, *S*_2_, *S*_3_ are the sampling matrices we use for different wavelength channels, and *H_m_(x,y)* represents the achromatic OAM‐selective hologram. This innovative approach leads to the successful construction of the achromatic OAM‐selective hologram. It is worth mentioning that in order to ensure that the reconstructed images of the three‐color channels in the same Fourier plane have the same size, we scaled the original images in advance, which is shown in Supplementary Note 3 and Supplementary Figure S3 (Supporting Information). The simulation results presented in Figure 2c demonstrate the reconstructed images from the achromatic OAM‐selective hologram at various depths when illuminated by OAM beams with *l*_re_ = −3 at three wavelengths. As shown, the holographic images encoded in the three wavelength channels can only be observed with high fidelity at the same depth, which is determined by the pre‐set focal length of the lens functions. To further validate the effectiveness of our method, we perform a pixel intensity analysis across the four selected pixels. Figure 2c shows the intensity distributions of these pixels across different depths, providing detailed insights into the spatial characteristics of the holographic output light beams. Additionally, we conducted comprehensive intensity distribution analysis to evaluate the performance of the proposed achromatic OAM‐selective hologram. Figure 2d highlights the significant improvement in the axial chromatic effect across the three different color channels (460, 532, and 633 nm). We normalized the four points selected in the white box with the maximum values of the three channels. The simulation results reveal that at a depth of d = 9 mm, the average normalized intensities across all three‐color channels simultaneously reach their maximum, thereby confirming the successful achievement of the axial achromatic effect for the three wavelengths. These results underscore the robustness and versatility of our method in enabling achromatic OAM‐selective holography across multiple color channels. (Crosstalk analysis of different color channels is provided in Figure S4, Supporting Information)

### 2.3 Experimental Characterization of Achromatic OAM‐Selective Hologram

**Figure 3:**
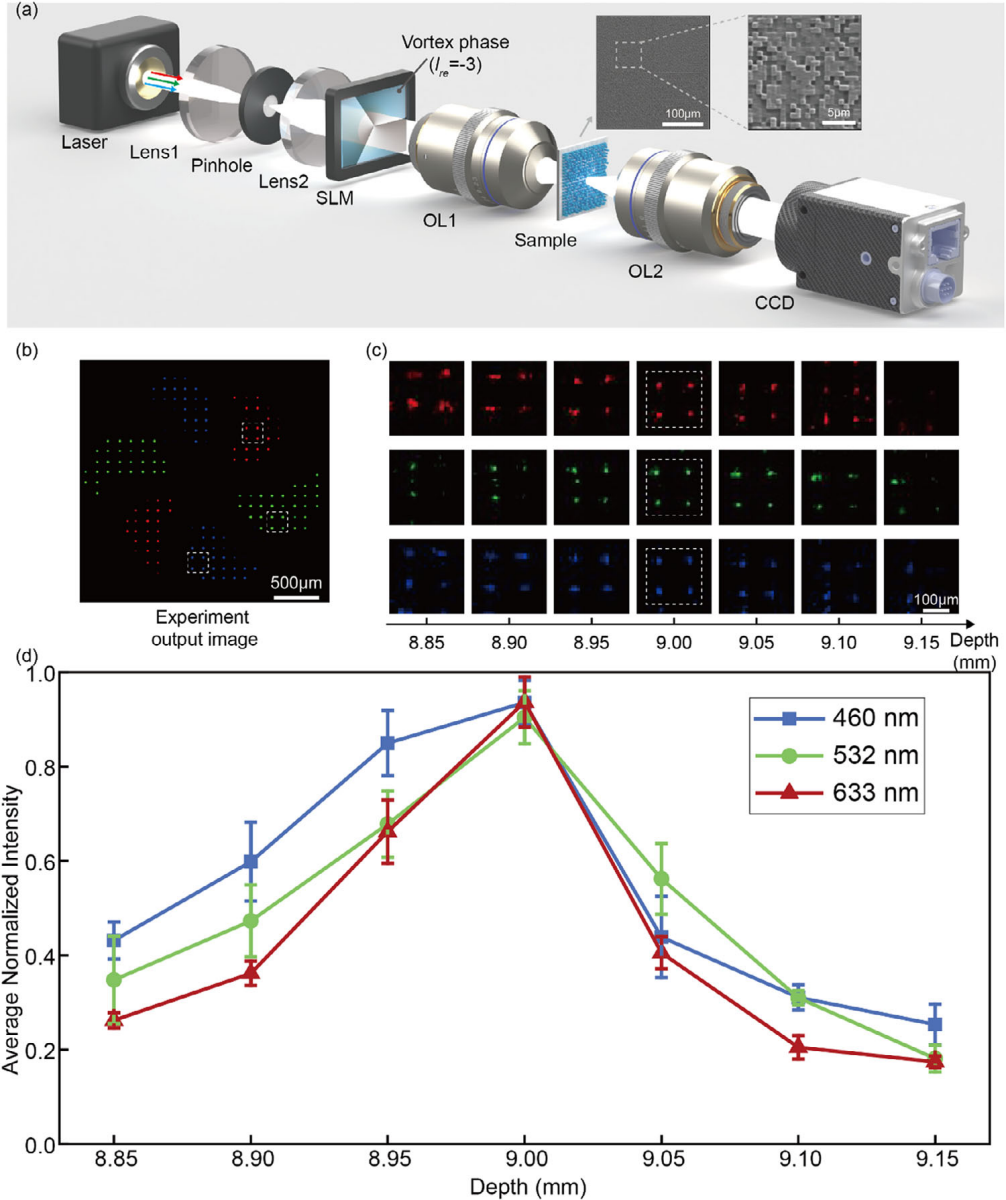
Experimental characterization of the optically digitalized achromatic OAM‐selective hologram. a) Schematic illustration of the optical setup. The insets reveal the microscope image and SEM image of the sample. b) Reconstructed color image at a pre‐set depth of 9 mm. c) Normalized intensity distributions of the four selected pixels labeled in the white dashed boxes across different depths. d) Quantitative analysis of the average normalized intensities of the four selected pixels in the white dashed boxes across different depths.

In the experiment, we successfully achieved the optically digitalization of a high‐resolution and polarization‐independent achromatic OAM‐selective hologram onto a silica substrate via TPL technology. The phase value of each pixel, *Φ*, was accurately encoded into a relative height map of pillars through the relationship Δ*Z = λΦ / 2π*Δ*n*, where Δ*n* represents the refractive index difference between the photoresist and air, and *λ* denotes the wavelength of the target modulated beams. To ensure precise control over the modulation, we considered three distinct 2π‐phase modulation heights for wavelengths of 633, 532, and 460 nm, each requiring separate optimization for optimal performance. The TPL process utilizing a Photonic Professional GT system (Nanoscribe GmbH) facilitated the fabrication of an achromatic OAM‐selective hologram with a feature size of 600 nm × 600 nm. Detailed operation procedures for this process are provided in Supplementary Note 5 (Supporting Information). The sample, with a total size of 276 µm × 276 µm, is shown in the insets of **Figure** **3**a.

Next, we characterize the optically digitalized achromatic OAM‐selective hologram using the optical setup depicted in Figure 3a (Supplementary Note 5 and Figure S5, Supporting Information). Our system employs a supercontinuum laser capable of generating laser beams with wavelengths of 633, 532, and 460 nm, either separately or simultaneously. After passing through the beam expansion system consisting of two convex lenses (JCOPTIX, OLC2401) and a pinhole, the horizontally polarized light was projected onto the helical phase plates embedded on the spatial light modulator (SLM, Holoeye GAEA‐2), producing vortex beams with topological charge *l_re_* = −3 at each wavelength. After imaging through two objective lenses (OL1 and OL2), the reconstructed OAM patterns were captured on a charge‐coupled device (CCD, Baslar, acA3088‐57uc). Notably, the position of the CCD could be adjusted along the light propagation direction to capture different imaging planes. As can be seen in Figure 3b, the color image with an SSIM of 0.75 can only be observed at the pre‐set depth of 9 mm. In Figure 3c, we further analyze the evolution of the intensity distributions of the pixels in the three white dashed boxes. For the reconstructed color image, we calculate its absolute efficiency (defined as the ratio of the energy of the reconstructed image to the energy of the input vortex beam at the corresponding wavelength channel and OAM channel), the absolute efficiency is 3.89% at 460 nm, 4.12% at 532 nm, and 3.72% at 633 nm.

The quantitative analysis of the average normalized intensity within these regions shows consistent overlap of the Fourier imaging planes across different wavelengths, which aligns with our theoretical ACA correction design (Figure 3d).

### 2.4 Experimental Demonstration of the Achromatic 3D OAM‐Multiplexing Holography

Based on the OAM sensitivity of the achromatic OAM‐selective hologram with ACA correction, we developed a method to achieve 3D multi‐color OAM‐multiplexing holographic encoding. The detailed design approach of the achromatic OAM‐multiplexing hologram is illustrated in Supplementary Note 6 and Figure S6 (Supporting Information).

**Figure 4:**
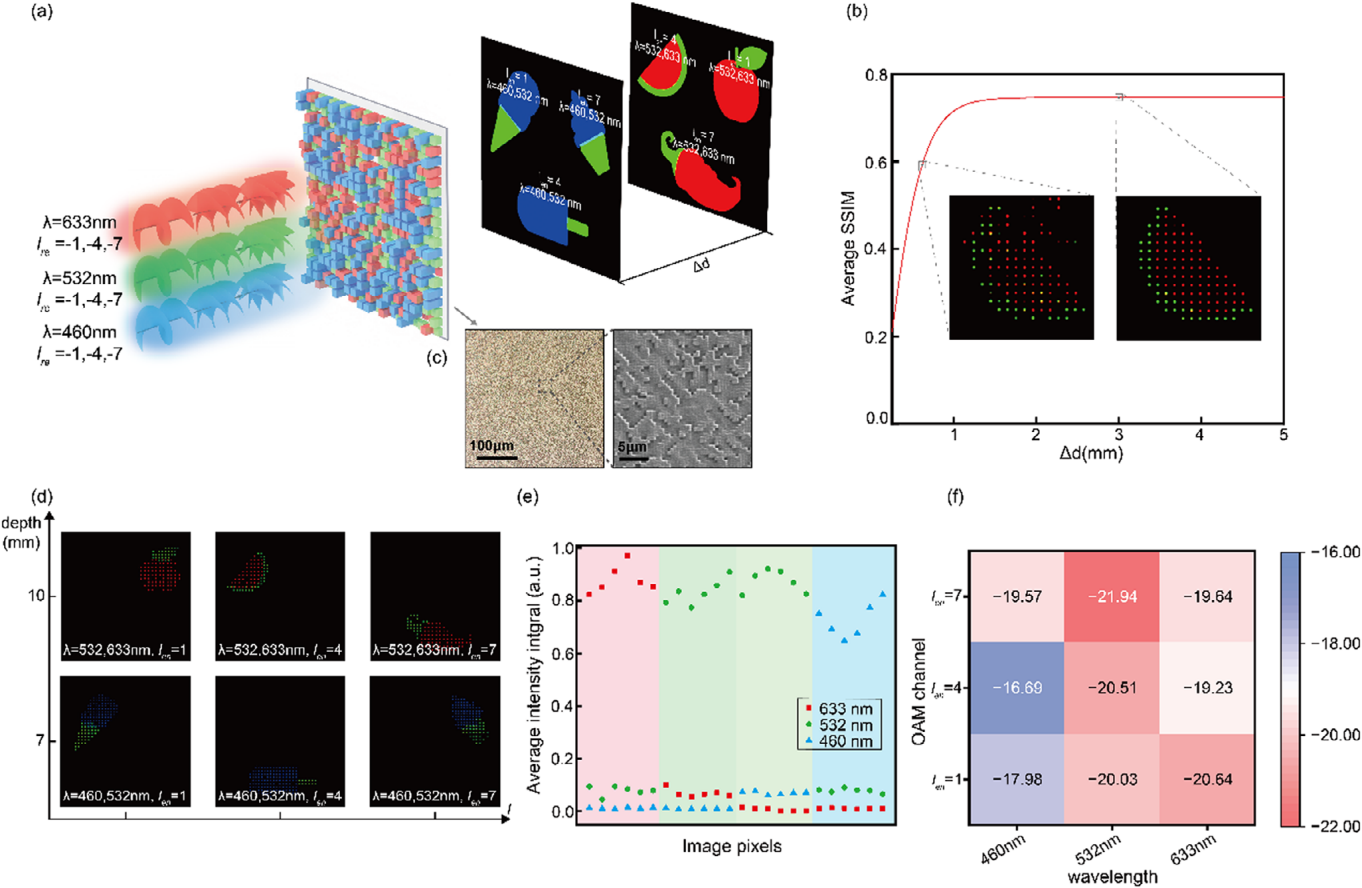
Experimental demonstration of the achromatic 3D OAM‐multiplexing holography. a) Schematic illustration of the double‐plane, 3‐color, OAM‐switchable holographic display. b) The relationship of the image reconstruction quality with the separation distance of imaging planes. The insets reveal two color reconstructed images when the spacing is 1 and 3 mm, respectively. c) The microscope and SEM images of the optically digitalized achromatic OAM‐multiplexing hologram. d) The reconstructive color images at distinct imaging depths by illuminating the optically digitalized achromatic OAM‐multiplexing hologram with OAM decoding beams of different colors. e) The modal crosstalk analysis for each image channel is based on selected pixels. f) The crosstalk matrix of wavelength ×OAM channels.

Here, six color images are divided into three groups of monochromatic images, further assigned to two imaging planes with a separation distance of Δ*d*. Within each group, the monochromatic images are encoded into three orthogonal OAM channels (*l_re_* = −1, −4, −7). After the ACA correction scheme involving the random spatial discretization and interleaving mentioned above, the achromatic OAM‐multiplexing hologram can be obtained (**Figure** **4**a). Notably, the separation distance between the two imaging planes Δ*d* is critical for image reconstruction quality, with optimal values exceeding 1.7 mm leading to consistent average SSIM values (≈0.76) across different spacing distances. The insets in Figure 4b reveal two color reconstructed images when the spacing is 1 and 3 mm, respectively.

To validate the feasibility of the achromatic 3D OAM‐multiplexing holography, we experimentally prepared the optically digitalized achromatic OAM‐multiplexing hologram using the TPL technology. To increase the spatial bandwidth product of the hologram, the hologram, with a total size of 408 µm × 408 µm and resolution of 680 × 680 into 3 × 3 parts, was segmented into 3×3 parts for stepwise processing. The microscope and SEM images of the sample are provided in Figure 4c. By illuminating the optically digitalized achromatic OAM‐multiplexing hologram with OAM decoding beams of different colors, six color images were successfully retrieved and displayed at the distinct imaging depths as illustrated in Figure 4d, compared with the target image, the average SSIM of the reconstructed image is ≈0.74. Furthermore, to enhance the signal‐to‐noise ratio (SNR) of the reconstructed multiplexed holographic images, we applied a mode‐selective aperture array with periodicity derived from the sampling array in the hologram design (Supplementary Note 7 and Figure S7, Supporting Information), and we obtained a reconstructed color image with SNR = 19.80 dB. This post‐processing step effectively suppressed noise and improved the clarity of the encoded data.^[^ ^29^ ^]^ To evaluate the performance of achromatic multi‐color OAM holography, we sampled part of Figure 4d from left to right and from top to bottom (the numeric labels in Figure S10 (Supporting Information) represent the arrangement order of the sampled pixels in Figure 4d), the average integrated intensity of the selected pixels is presented in Figure 4e, demonstrating the effectiveness of the proposed ultra‐compact holography system in achieving double‐plane, 3‐color, OAM‐switchable display. Furthermore, we demonstrated the crosstalk matrix of wavelength × OAM channels in Figure 4f. Notably, the average crosstalk is defined as the ratio of the intensity over all unwanted wavelength and OAM channels to the intensity in the target wavelength and OAM channel.

## 3 Conclusion

In summary, our work presents a significant breakthrough in achromatic 3D multi‐color OAM holography, effectively solving the critical issue of ACA in conventional Fourier lens‐based OAM holographic systems. By implementing a spatial multiplexing scheme on monochromatic OAM‐dependent holograms encoded with wavelength‐specific lens functions, we achieve precise spatial coincidence control across multiple wavelengths in the spatial frequency domain. Our innovative approach utilizes an optically digitalized hologram with a subwavelength pixel size of 600 nm, achieved through advanced TPL‐based nano‐printing technology, which enables high spatial bandwidth products. As a result, we successfully achieved the double‐plane (along the light direction), 3‐color (633, 532, and 460 nm), and 3‐channel (OAM orders *l_en_* = 1, 4, 7) holographic display with ACA correction. Our method represents a significant advancement over existing approaches, as it effectively mitigates ACA through innovative spatial multiplexing utilizing polarization‐independent diffractive optical elements, thereby eliminating the need for complex polarization‐optical‐element setups. This simplification not only reduces system complexity but also enhances the efficiency and reliability of the holographic process, which not only enhances the performance of achromatic 3D multi‐color OAM holographic displays but also opens new avenues for future research, enhancing the capabilities of lightweight AR/VR wearables in OAM‐addressable dynamic visual effects and multi‐user personalized content. Addressing potential limitations such as wavelength channel number and the level of crosstalk within the spatial multiplexing scheme could further optimize the method's performance,^[^ ^49^ ^]^ paving the way for more efficient and versatile applications. In conclusion, by combining the advantages of wavelength multiplexing and OAM multiplexing, our method provides a powerful tool for achieving high‐quality, high‐information capacity holographic systems. These results have broad implications for the development of achromatic 3D optical systems, paving the way for innovative technologies in optical holography.

## 4 Experimental Section

### Computational Platform

All the calculations were performed on a personal desktop with an Intel(R) Core(TM) i5‐10500 CPU @ 3.10 GHz, and 12GB of RAM, running the Windows 10 for Workstations. The code was written, compiled, and run in the MATLAB R2022a software.

### Fabrication of the Holograms

Both OAM‐selective holograms and OAM‐multiplexing holograms mentioned in this paper were fabricated for experimentation using the same nanofabrication process. The details of the processing are available in Supplementary Note 4 (Supporting Information).

### Experimental Characterization

All experimental characterization such as the laser, optical element, and CCD are available in Supplementary Note 5 and Figure S5 (Supporting Information).

### Statistical Analysis

The SNR and the SSIM were introduced as the objective image quality function to evaluate the reconstruction of achromatic OAM‐dependent holograms. SNR and SSIM are widely used metrics to measure the quality of a compressed or reconstructed image or video signal. Essentially, SNR measures the similarity between two reconstructed scenes by comparing the signal magnitude and noise, while SSIM evaluates their structural information, such as luminance, contrast, and structure. They are respectively expressed as:

(4) $$SNR\left(dB\right)=10\cdot \log_{10}\left(\frac{I_{r}}{I_{n}}\right)$$

where SNR is calculated by taking the logarithm (base10) of the reconstructed image signal intensity *I_r_* and noise intensity *I_n_* SNR is often used to quantify the quality of a reconstructed signal by measuring the ratio of signal strength to noise. The higher the SNR value, the better the image quality.

(5) $$SSIM=\left(\frac{2\cdot \mu_{r}\cdot \mu_{t}+C_{1}}{\mu_{r}^{2}+\mu_{t}^{2}+C_{1}}\right)\cdot \left(\frac{2\cdot \sigma_{r,t}+C_{2}}{\sigma_{r}^{2}+\sigma_{t}^{2}+C_{2}}\right)$$

where, means (*μ_r_*, *μ_t_*) represent the average intensities of the reference target image and the OAM holography image patches, calculated as:

(6) $$\mu_{r}=\frac{1}{N}\sum_{i=1}^{N}r_{i}$$

(7) $$\mu_{t}=\frac{1}{N}\sum_{i=1}^{N}t_{i}$$

where *N* is the number of pixels in the local window.

Standard Deviations (*σ_r_*, *σ_t_*) quantify the contrast of the patches in target and reconstructed OAM holography image, derived from the unbiased estimators:

(8) $$\sigma_{r}=\sqrt{\frac{1}{N-1}\sum_{i=1}^{N}{\left(r_{i}-\mu_{r}\right)}^{2}}$$

(9) $$\sigma_{t}=\sqrt{\frac{1}{N-1}\sum_{i=1}^{N}{\left(t_{i}-\mu_{t}\right)}^{2}}$$

Cross‐covariance (*σ_r,t_*) measures the linear correlation between the two patches:

(10) $$\sigma_{r,t}=\frac{1}{N-1}\sum_{i=1}^{N}\left(r_{i}-\mu_{r}\right)\left(t_{i}-\mu_{t}\right)$$

The calculations were performed over local windows across the entire image, as per the SSIM methodology. The constants *C*_1_ and *C*_2_ are used for stability. SSIM assesses the structural information of two images, including luminance, contrast, and structure. It produces a value between 0 and 1, and the larger the value, the smaller the image distortion and the more similar the two images are.

## Conflict of Interest

The authors declare no conflict of interest.

## Author Contributions

X.F. conceived the idea; X.F. and B.L. supervised the project; H.S. designed and fabricated the nano‐printing achromatic 3D color OAM‐multiplexing hologram; H.S. recorded and processed the experimental results; Y.Y. and Y.B. helped the visualization of the results; X.F. and B.L. wrote the manuscript with the contributions of all authors.

## Supporting information



Supporting Information

## Data Availability

The data that support the findings of this study are available from the corresponding author upon reasonable request.
