# Supplementary material for: Achromatic 3D Multi‐Color Orbital Angular Momentum Holography
Source: Adv Sci (Weinh). 2025 Apr 24;12(26):2503488. doi: 10.1002/advs.202503488 (PMC12245109; doi:10.1002/advs.202503488)
Supplement: Supplementary file 1 — Supporting Information [file ADVS-12-2503488-s001.docx]

Supplementary Materials for

Achromatic 3D multi-color orbital angular momentum holography

*Hang Su ^1,2^, Baoli Li ^1,2*^, Yike Bai ^1,2^, Yiqi Ye ^1,2^, Yibo Dong ^1,2^ and Xinyuan Fang ^1,2*^*

^1^School of Artificial Intelligence Science and Technology, University of Shanghai for Science and Technology, Shanghai, 200093, China.

^2^Institute of Photonic Chips, University of Shanghai for Science and Technology, Shanghai, 200093, China.

*Corresponding author. Email: xinyuan.fang@usst.edu.cn; [baolili@usst.edu.cn](mailto:baolili@usst.edu.cn)

**This PDF file includes:**

Supplementary Note 1 to 7

Supplementary Table S1

Supplementary Figures S1 to S11

**Supplementary Note 1: Characterization of Orbital angular momentum (OAM)-dependent sampling constants in the spatial frequency domain.**

In order to reduce destructive interferences of the OAM pixels, we selected the vortex light diameter corresponding to 30% of the maximum amplitude as the minimum sampling distance of the sampling array. Therefore, the minimum sampling spacing corresponding to the three wavelengths (633nm, 532nm and 460nm) and two Fourier plane depths (7mm and 10mm) used in the experimental demonstration of achromatic OAM-multiplexed holography is shown in Supplementary Figure S1. In order to ensure the resolution and signal-to-noise ratio (SNR) of the reconstructed image, we selected the sampling spacing of red light at different OAM orders as the benchmark and designed the sampling array to sample the target image.

**Supplementary Note 2: Principle of the randomly spatial discretization and interleaved.**

In order to reduce the crosstalk between channels of different wavelengths, we design the holograms of the three wavelengths individually and spatially multiplex them using a random sampling method. The sampling matrix is obtained by the method of Supplementary Figure S2. For an N*N matrix, it is discretized into three sampling matrices by adjusting the probability of any pixel appearing in the three channels. The results shown in Supplementary Figure S2a are obtained when the probability of occurrence is equal in all three channels, i.e., Pr : Pg : Pb = 1:1:1, when the number of sampling points in the three sampling matrices is approximately equal. Furthermore, this system allows for precise control over the modulation efficiency of individual wavelength channels by precisely adjusting the corresponding probability parameter.

Supplementary Figure S2b shows the process of discrete sampling and spatial multiplexing of the three wavelength channel holograms using a random sampling matrix.

**Supplementary Note 3: Horizontal scaling for color holographic reconstruction.**

Since reconstruction at different wavelengths produces images of different sizes, resulting in no way to form a perfect color image, it is necessary to process the original image before encoding to ensure that the reconstructed holographic images at different wavelengths have the same size and the same dot spacing. For the original input image (as shown in the upper part of Supplementary Figure S3), under the simulation and experimental conditions in this paper, the target images at wavelengths of 633 nm, 532 nm and 460 nm are scaled according to the ratios of 0.850, 1 and 1.156, respectively, and the scaled images are sampled according to the same ratios of the spacing, and the preprocessed images are shown in the middle part of Supplementary Figure S3. By adjusting the image size and sampling spacing at different wavelengths, the holograms are able to obtain holograms with consistent size and dot spacing at different reconstructed wavelengths without the need for additional optical scaling, which is crucial for realizing color OAM multiplexed holography.

**Supplementary Note 4: The fabrication of achromatic 3D multi-color orbital angular momentum hologram using two-photon polymerization lithography(TPL).**

The fabrication of achromatic 3D multicolor orbital angular momentum holograms was performed using a commercial lithography system (Photonic Professional GT, Nanoscribe GmbH) equipped with a 780 nm, 80 MHz femtosecond laser. The system employed an immersion galvanometer scanning mode configuration for printing, utilizing a high numerical aperture objective (Plan-Apochromat 63x/1.40 Oil DIC, Zeiss) and IP-Dip photoresist. The maximum heights corresponding to 2π phase modulation for the 633 nm, 532 nm, and 460 nm channels were 1.266 μm, 1.064 μm, and 0.920 μm, respectively. During processing, the slice distances (longitudinal laser movement steps) were set to 0.158 μm, 0.133 μm, and 0.12 μm, corresponding to phase modulations of 1/4π for the 633 nm, 532 nm, and 460 nm lasers, respectively. The lateral laser movement step was uniformly set to 0.1 μm. To achieve optimal results, the laser power and scanning speed were optimized to 50 mW and 10,000 μm/s, respectively. Post-printing, to ensure that the residual photoresist was removed while preserving the processed structure, the sample was immersed in propylene glycol 1-monomethyl ether 2-acetate for 30 minutes for development, followed by a 5-minute wash in Isopropanol. Finally, the sample was evaporated and dried in air.

**Supplementary Note 5: Experimental setup for achromatic 3D color OAM multiplexed holograms.**

In the characterization light path of achromatic holograms (Supplementary Figure S5), we first use a continuous spectrum laser (YSL, AOTF) as the light source, which can achieve continuous spectral output from 400 nm to 700 nm, basically covering the entire visible light band. Next, a half-wave slice (HWP, Thorlabs, WPH05M-532) and a polarizing beamsplitting cube (PBS, Thorlabs, PBS251) are used to achieve continuous modulation of the optical power, while the PBS adjusts the polarization state of the input light to be horizontal, ensuring that the subsequent spatial light modulator has maximum modulation efficiency. The incident light passes through a spatial filtering system consisting of a lens (JCOPTIX, OLC2401) and a pinhole (Thorlabs, P50K) and a beamsplitter cube (BS, Thorlabs, BS013) before being incident on a spatial light modulator (SLM, Holoeye, GAEA-2), where a spiral phase of a specific order is added to the SLM to select the information of the different OAM channels in the hologram. It is worth noting that, as different wavelengths of light enter the experimental system sequentially, the SLM needs to adjust its modulation curves for different incident wavelengths to ensure the same modulation efficiency for different wavelengths. Next, the vortex beam generated by the SLM is irradiated onto the hologram sample by narrowing it through the objective lens 1 (SOPTOP, PLAN20X), and the results of the holographic display are observed and recorded through the objective lens 2 (SOPTOP, PLAN20X) and the charge-coupled device (CCD, Baslar, acA3088-57uc), and the information of different depth planes is observed by moving the front and back position of the CCD.

**Supplementary Note 6: Design of achromatic color 3D OAM multiplexed hologram.**

In order to reconstruct the color image information distributed with three OAM channels in two depth planes, we first discretize the target image into three wavelength channels, and then introduce the method of obtaining the multiplexed holograms using the green channel as an example. First, we utilize the sampling dot matrix to sample the image information within this channel, followed by obtaining the OAM-preserved hologram by inverse Fourier transform. Then, the six images were divided into three groups and multiplexed after superimposing the spiral phases of *l_en_* = 1, 4, 7 to obtain two OAM-selective holograms. In order to reconstruct the information of these two holograms in different depth planes, two lens phases with different focal lengths were superimposed on the OAM- selective holograms and the two holograms were combined. Finally, the holograms obtained from the three wavelength channels are spatially multiplexed using the random sampling method introduced in Supplementary Note 2 to obtain achromatic 3D OAM-multiplexing holograms. Depending on the vortex beam irradiation carrying a specific topological charge, this hologram can be reconstructed in two depth planes with switchable color image information.

**Supplementary Note 7: The process of post-processing using mode-selective aperture array.**

In order to improve the signal-to-noise ratio of reconstructed holographic images in achromatic 3D OAM-multiplexing holography, a mode-selective aperture array is utilized in the post-processing of the output results. We designed a pattern selection aperture array with the same parameters as the initial color OAM hologram (including size, pixel size and sampling distance), and processed the hologram corresponding to the pattern selection aperture array into a separate structure using TPP. The hologram, experimental results and working principle of the mode selection aperture array are shown in Supplementary Figure S7.

In order to align the mode-selective aperture array with the reconstructed hologram image, we intentionally fabricate the mode-selective aperture array hologram structure adjacent to the OAM multiplexing hologram on the same substrate. After capturing the reconstructed light field with a charge coupled device (CCD), we pinpointed the intensity peak coordinates of the reconstructed focal spots. By superimposing these coordinates as fiducial markers, we performed lateral mechanical displacement adjustments to the glass substrate until the intensity peaks of the aperture array spatially overlapped with those of the reconstructed focal spots.

**Supplementary Table S1:** Comparison of performance indexes of 3D multi-color OAM holography.

|  | Total number of channels | Number of color channels | Number of 3D planes | Number of OAM channels | Achromatic capability |
| --- | --- | --- | --- | --- | --- |
| Ref.[1] | 4 | 1 | 10 | 4 | No |
| Ref.[2] | 5 | 1 | 2 | 5 | No |
| Ref.[3] | 8 | 2 | 1 | 4 | No |
| Ref.[4] | 6 | 2 | 1 | 3 | No |
| This work | 6 | 3 | 2 | 3 | Yes |

**
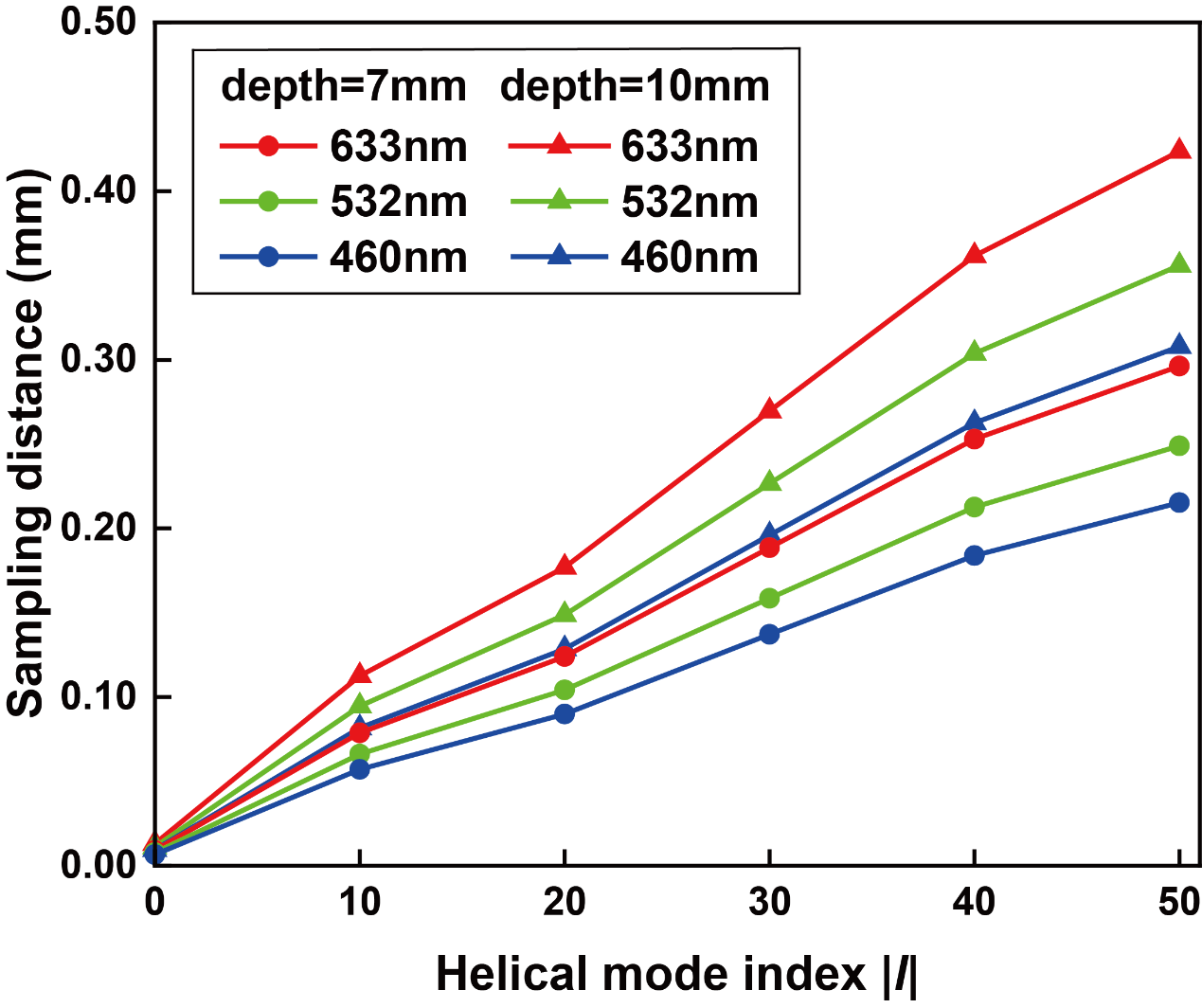
**

**Supplementary Figure S1.** Numerical characterization of OAM pixel size as a function of helical mode index |*l*| at different wavelength and depth.


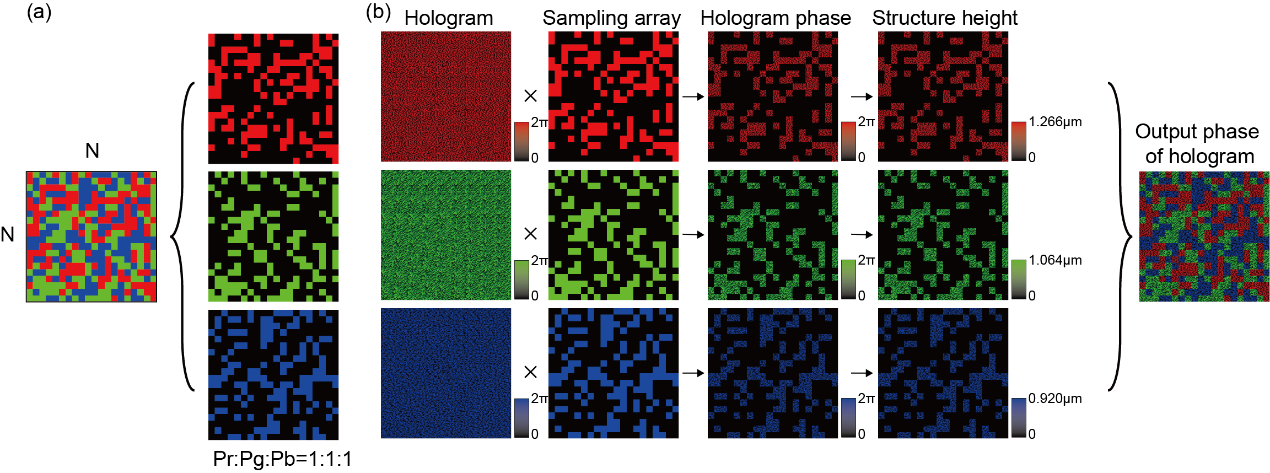


**Supplementary Figure S2.** Principle of random sampling of holograms. (a) The N*N matrix is discretized into three sampling matrices with probability Pr : Pg : Pb = 1:1:1. (b) Sampling and compositing the holograms of the three wavelength channels using the sampling matrices in (a).


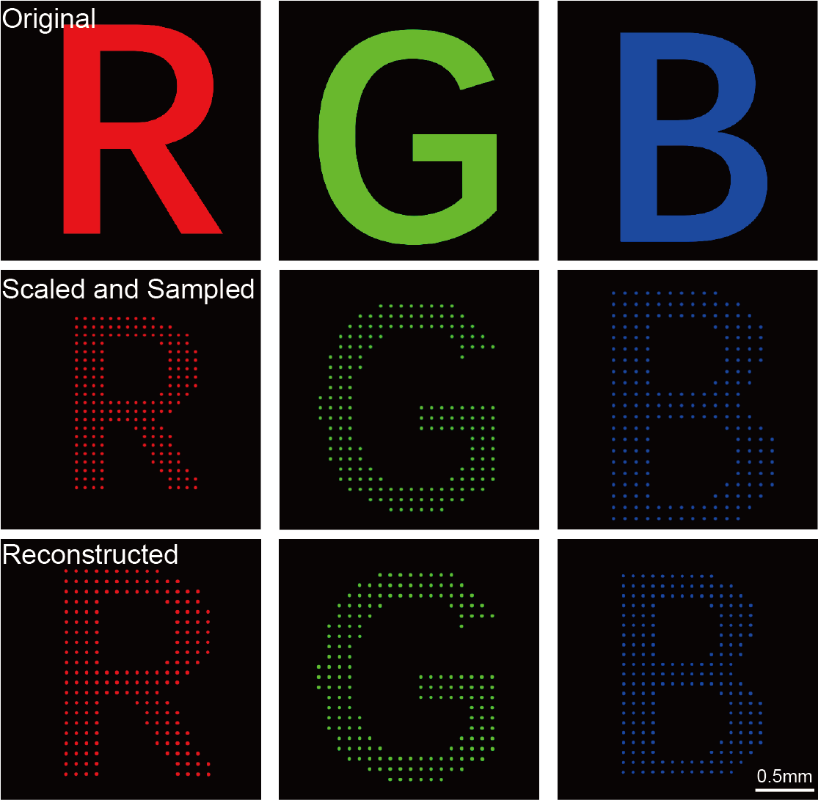


**Supplementary Figure S3.** Original images (upper), scaled and sampled images (middle) and reconstructed images (lower) of alphabet R, G and B, corresponding to the wavelength of 633, 532 and 460 nm, respectively.


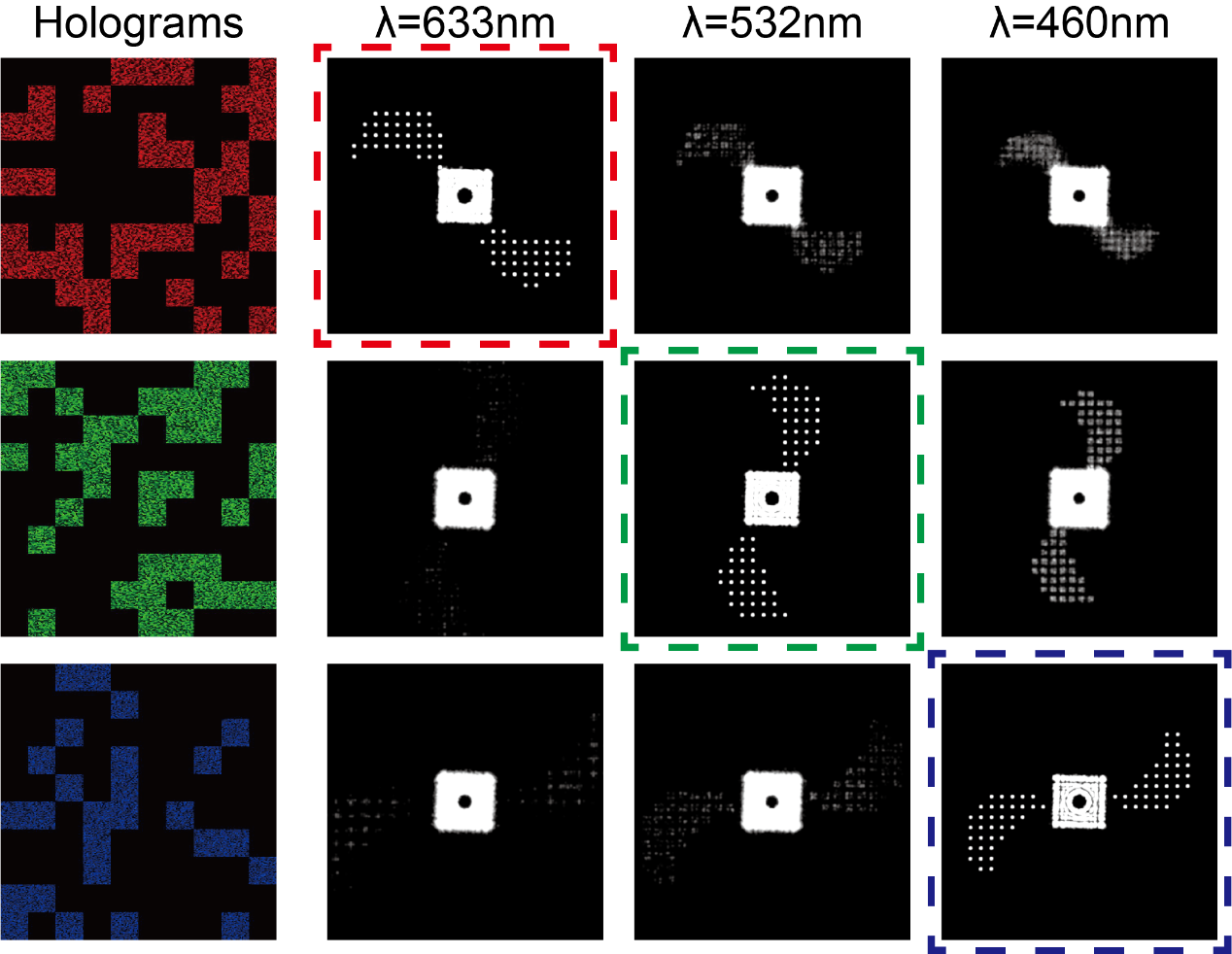


**Supplementary Figure S4.** Wavelength crosstalk analysis of the achromatic multi-color OAM-selective hologram. Each discrete achromatic OAM-selective hologram is illuminated by a vortex beam of 633 nm, 532 nm, and 460 nm with topological charge *l_re_* = -3. The crosstalk between different wavelength channels is defined as the ratio of the intensity in the desired wavelength channel to the sum of the intensities in all other unwanted wavelength channels. Through quantitative analysis of the intensity at predetermined positions, the inter-channel crosstalk for the achromatic polychromatic OAM-selective hologram was measured to be approximately -29.68 dB.


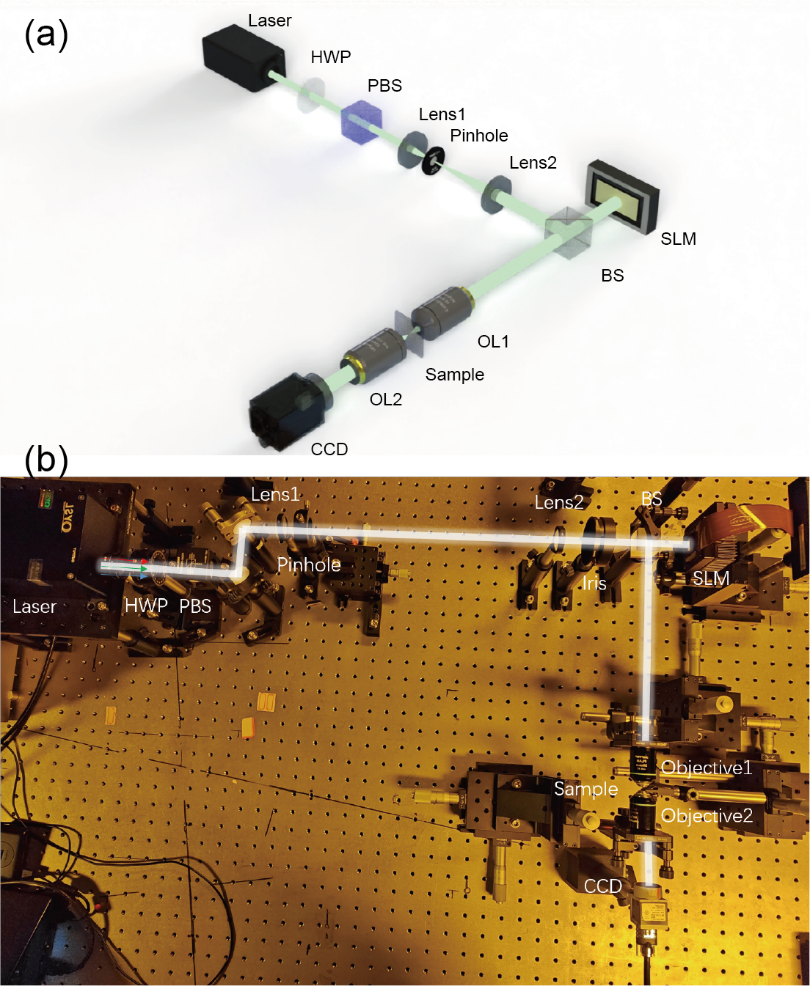


**Supplementary Figure S5.** Experimental setup for achromatic 3D color OAM-multiplexing holograms. (a) Schematic diagram of the experimental setup. (b) Photograph of the experimental system.


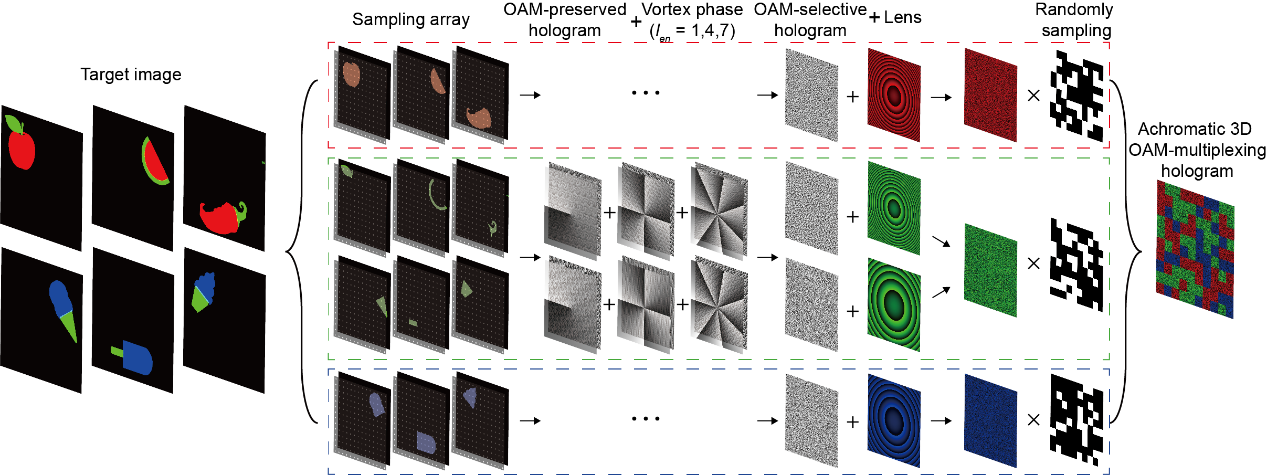


**Supplementary Figure S6.** Design principles of achromatic color 3D OAM-multiplexing holograms.


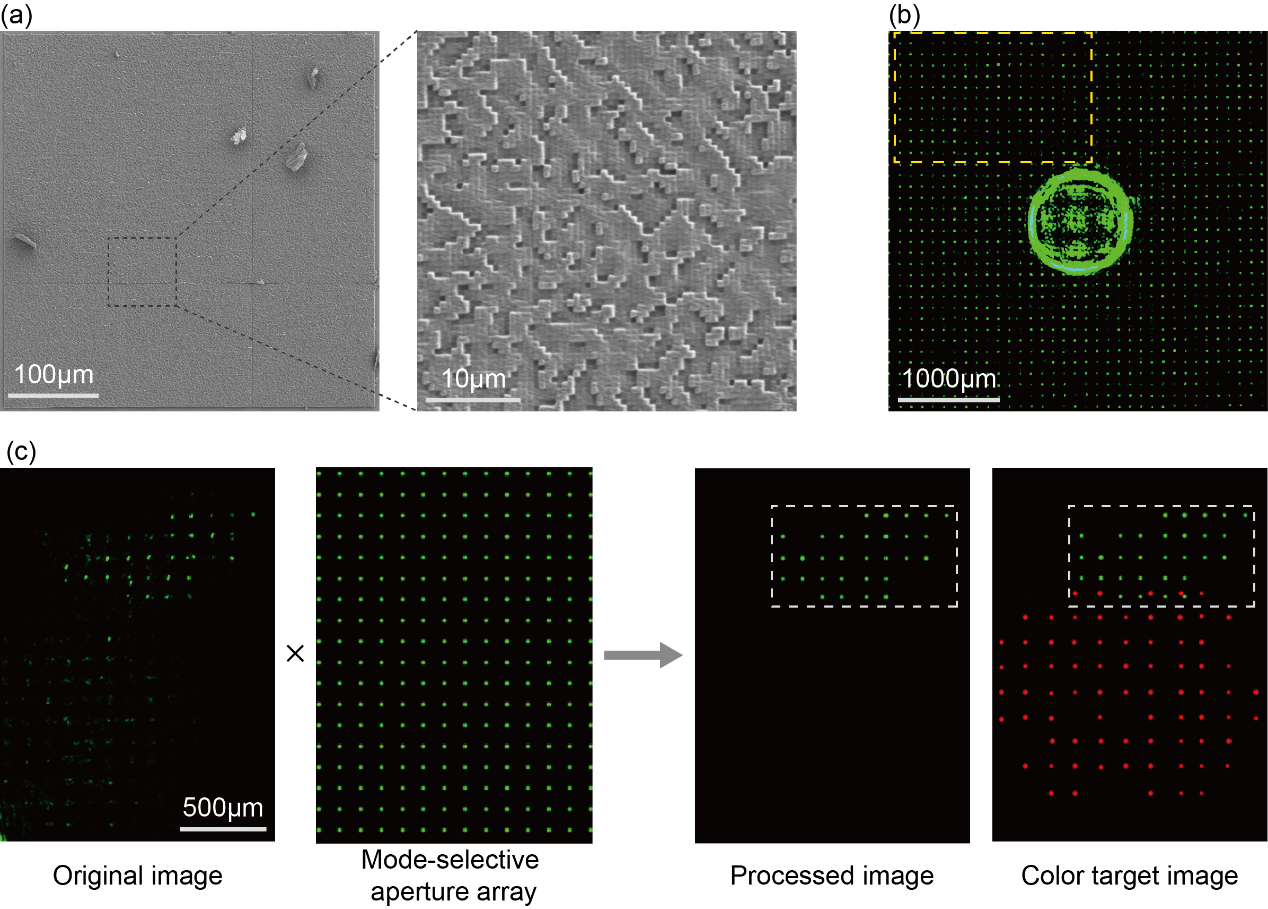


**Supplementary Figure S7.** The post-processing process of the reconstructed images using mode-selective aperture array. (a) SEM of digitalized hologram for the mode-selective aperture array. (b) Experiment results of the 2D spot array from the hologram to obtain the mode-selective aperture array, the yellow dotted box represents the target imaging area. (c) The detailed process of mode selection to improve the SNR.


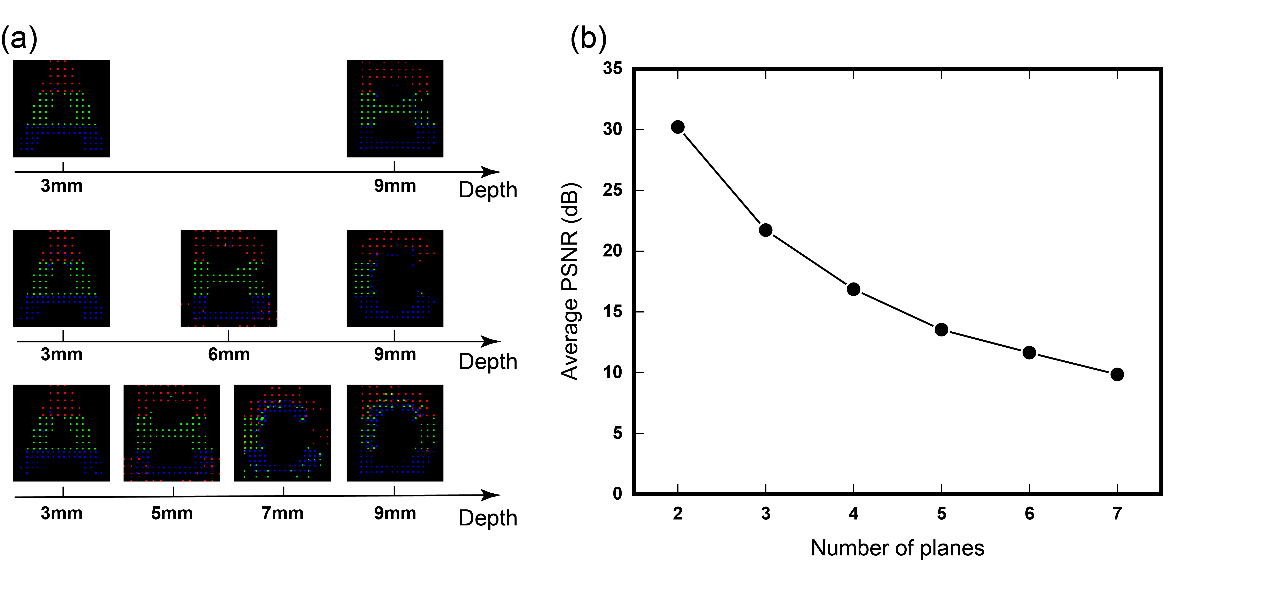


**Supplementary Figure S8.** The reconstruction results of achromatic 3D multi-color OAM holography in more planes. (a) The holographic color images reconstructed in 2, 3, and 4 planes. (b) The relationship of the Average PSNR with the with the number of image planes.


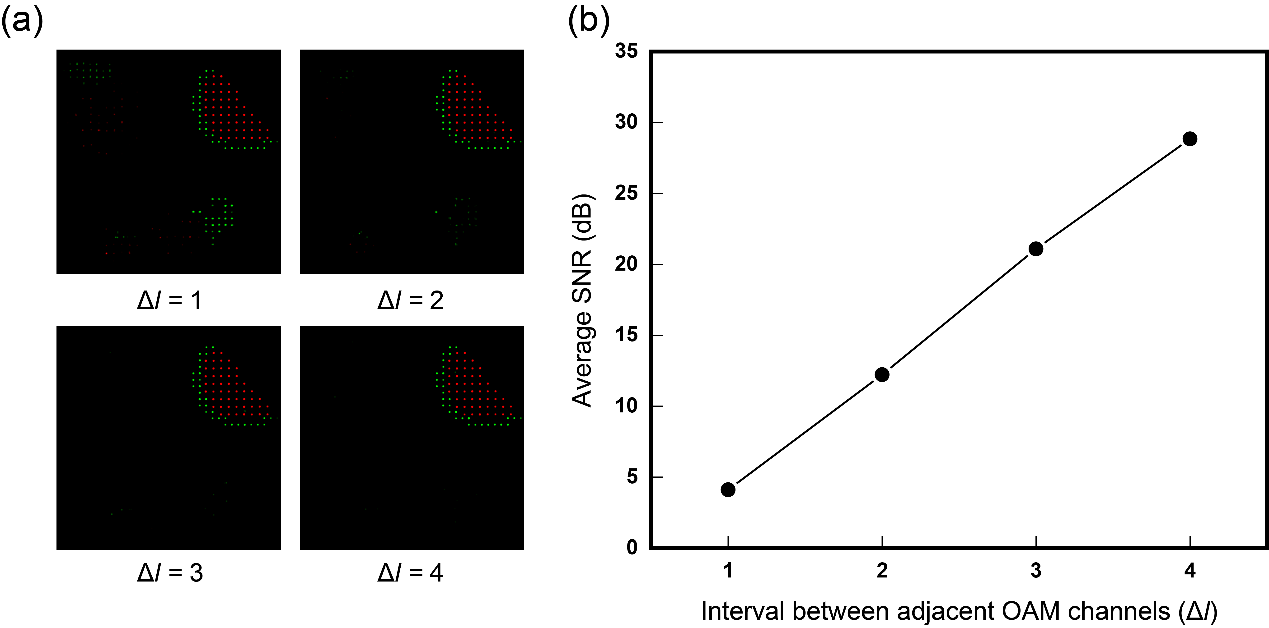


**Supplementary Figure S9.** The reconstruction holographic images encoded by different OAM channel intervals. (a) The holographic color images reconstructed with different OAM intervals. (b) The relationship of the Average SNR with the adjacent OAM channel interval Δ*l*.


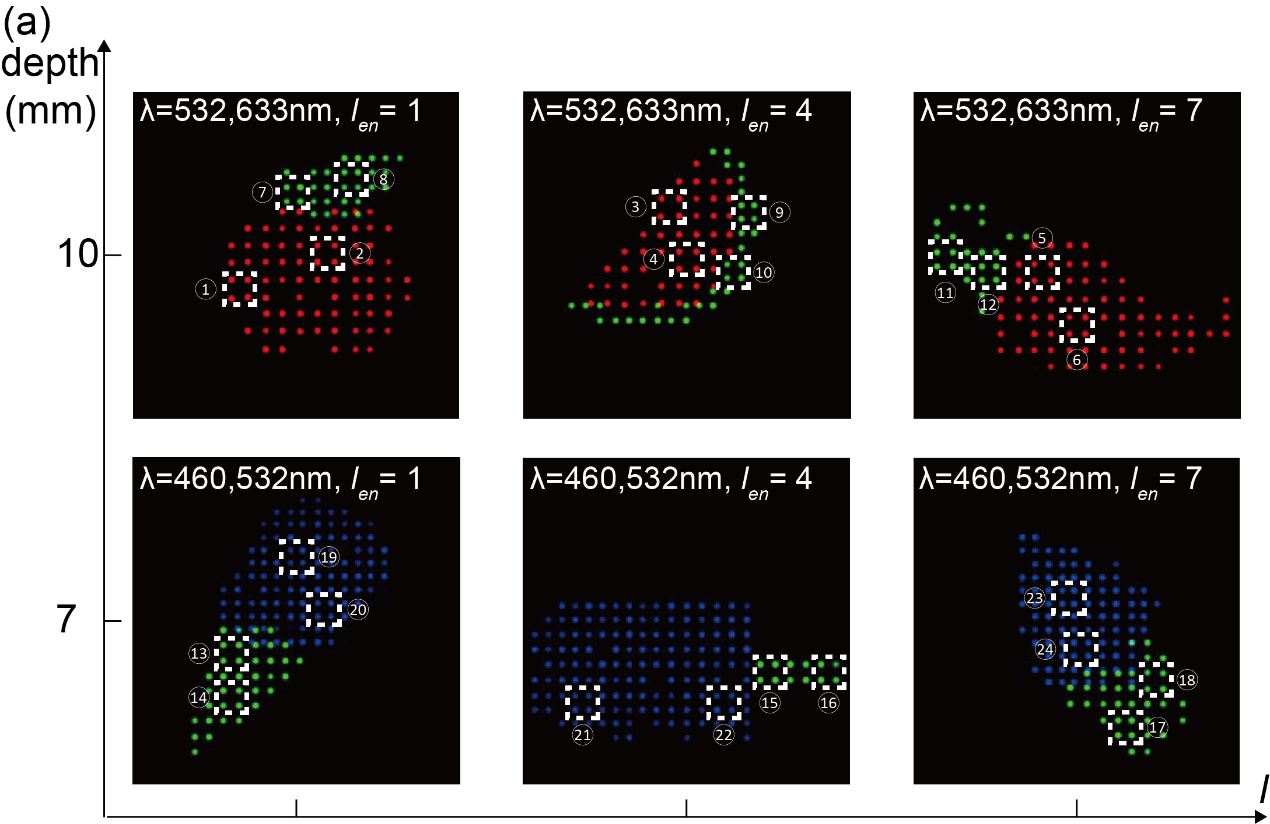


**Supplementary Figure S10.** Reconstructed color images at different magnified imaging depths and selected sampling regions


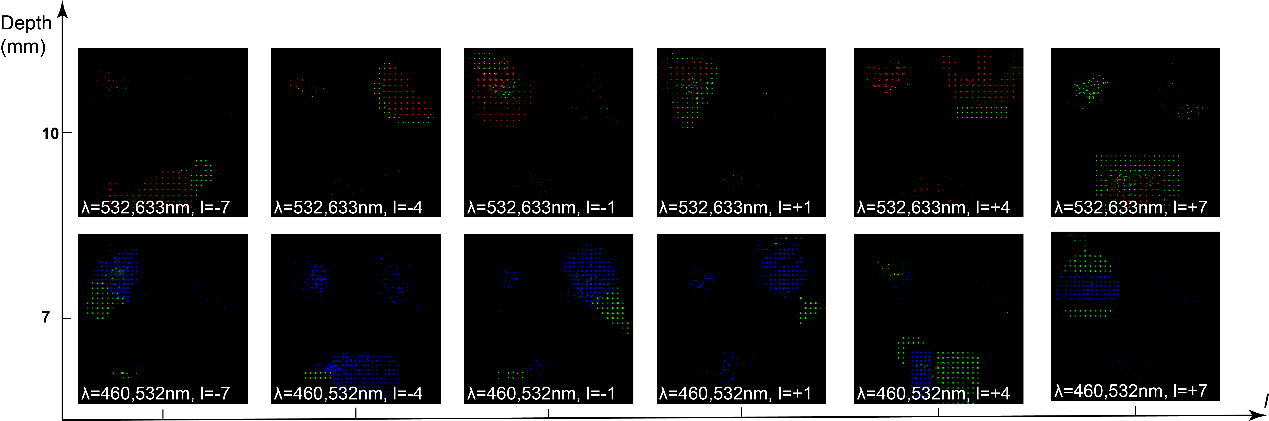


**Supplementary Figure S11.** Achromatic 3D OAM-multiplexing holography with spatial position overlap
